# Solution Structure of the Tandem Acyl Carrier Protein Domains from a Polyunsaturated Fatty Acid Synthase Reveals Beads-on-a-String Configuration

**DOI:** 10.1371/journal.pone.0057859

**Published:** 2013-02-28

**Authors:** Uldaeliz Trujillo, Edwin Vázquez-Rosa, Delise Oyola-Robles, Loren J. Stagg, David A. Vassallo, Irving E. Vega, Stefan T. Arold, Abel Baerga-Ortiz

**Affiliations:** 1 Department of Biochemistry, University of Puerto Rico – Medical Sciences Campus, San Juan, Puerto Rico; 2 Department of Biology, University of Puerto Rico – Rio Piedras Campus, San Juan, Puerto Rico; 3 Department of Biochemistry and Molecular Biology and Center for Biomolecular Structure and Function, The University of Texas MD Anderson Cancer Center, Houston, Texas, United States of America; 4 Division of Biological and Environmental Sciences and Engineering, King Abdullah University of Science and Technology, Thuwal, Saudi Arabia; University of Oulu, Finland

## Abstract

The polyunsaturated fatty acid (PUFA) synthases from deep-sea bacteria invariably contain multiple acyl carrier protein (ACP) domains in tandem. This conserved tandem arrangement has been implicated in both amplification of fatty acid production (additive effect) and in structural stabilization of the multidomain protein (synergistic effect). While the more accepted model is one in which domains act independently, recent reports suggest that ACP domains may form higher oligomers. Elucidating the three-dimensional structure of tandem arrangements may therefore give important insights into the functional relevance of these structures, and hence guide bioengineering strategies. In an effort to elucidate the three-dimensional structure of tandem repeats from deep-sea anaerobic bacteria, we have expressed and purified a fragment consisting of five tandem ACP domains from the PUFA synthase from *Photobacterium profundum*. Analysis of the tandem ACP fragment by analytical gel filtration chromatography showed a retention time suggestive of a multimeric protein. However, small angle X-ray scattering (SAXS) revealed that the multi-ACP fragment is an elongated monomer which does not form a globular unit. Stokes radii calculated from atomic monomeric SAXS models were comparable to those measured by analytical gel filtration chromatography, showing that in the gel filtration experiment, the molecular weight was overestimated due to the elongated protein shape. Thermal denaturation monitored by circular dichroism showed that unfolding of the tandem construct was not cooperative, and that the tandem arrangement did not stabilize the protein. Taken together, these data are consistent with an elongated beads-on-a-string arrangement of the tandem ACP domains in PUFA synthases, and speak against synergistic biocatalytic effects promoted by quaternary structuring. Thus, it is possible to envision bioengineering strategies which simply involve the artificial linking of multiple ACP domains for increasing the yield of fatty acids in bacterial cultures.

## Introduction

**Figure 1:**
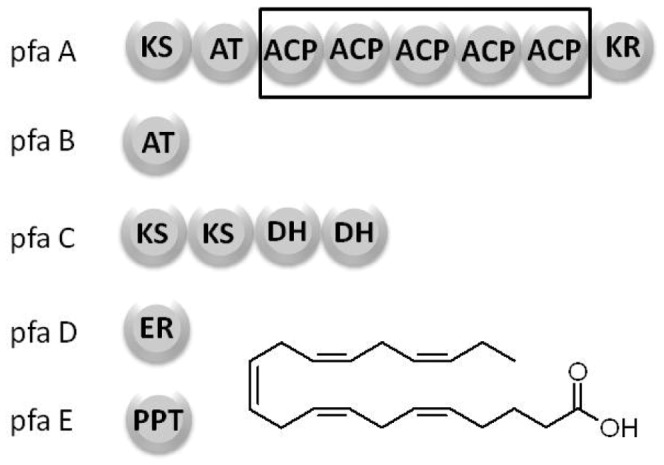
The polyunsaturated fatty acid synthase domain structure. A total of five genes are required for the production of PUFAs: pfaA contains a beta-ketoacyl synthase (KS), an acyltransferase (AT), five tandem acyl carrier proteins (ACP) and a ketoreductase (KR) domain. pfaB consists of a single AT. pfaC contains two KS domains and two tandem dehydratase (DH) domains, pfaD consists of a single enoyl reductase (ER) domain. pfaE consists of a phosphopantetheinyl transferase (PPTase).

Polyunsaturated fatty acids (PUFAs) are made by deep-sea bacteria employing an anaerobic mechanism that involves a polyketide synthase (PKS)-like multienzyme [1], [2]. In this multienzyme system, a total of five genes have been found to be required for the production of PUFA (Figure 1): *pfaA* which contains sequences corresponding to a ketoacyl synthase (KS) domain, an acyltransferase (AT) domain, a stretch containing multiple acyl carrier protein (ACP) domains and finally a ketoreductase (KR) domain; *pfaB* consists of a single AT domain; *pfaC* contains two KS homology domains and two DH domains; *pfaD* contains a single enoyl reductase (ER) domain and *pfaE* encodes for a phosphopantetheinyl transferase which is typically required for the activation of the ACP domains [3], [4]. While some work has been carried out on individual activities within this multienzyme system, the actual mechanism by which the enzymes act in concert to produce PUFAs has not been elucidated.

ACP domains fulfill an important function in the biosynthesis of fatty acids and polyketides. They are typically small, monomeric proteins with a three-dimensional fold corresponding to a four-helix bundle. ACP domains are generally considered scaffolds for the covalent attachment of acyl substrates and intermediates along the biosynthesis pathway. In fact, all modifications on the fatty acyl intermediates and all the elongation cycles that lead to the production of fatty acids and polyketides, take place while the intermediates are covalently attached to the ACP domain.

**Figure 2:**
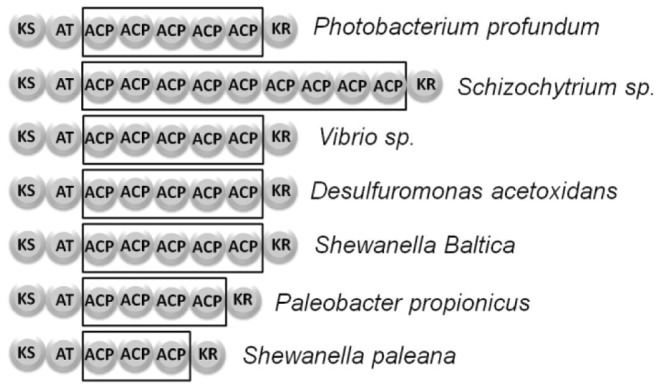
Evolutionary conservation of the tandem ACP domain. While the tandem ACP arrangement is rare among most PKS enzyme systems, it is a defining feature of the marine PUFA synthase multienzymes. Most PUFA producers contain between 2–9 ACP domains in tandem.

The ACP domains in the PUFA synthase clusters are arranged in tandem within the *pfaA* multidomain protein. This rare organization of enzyme domains in the PUFA synthase multienzyme is globally conserved among organisms that produce PUFAs in deep-sea environments [4], [5]. Numerous species of *Shewanella*, *Psychromonas* and *Nostoc* have been found to produce fatty acids in very high yields and to contain PUFA synthase genes organized as outlined in Figure 1. Although the general organization of domains in the PUFA gene cluster is conserved in different organisms, there is one area of variability: the number of ACP domains repeated in tandem along the sequence of *pfaA,* which tends to oscillate between 2 and 9 copies (Figure 2).

One possible explanation for the persistence of the tandem ACP throughout the evolution of PUFA synthases is that the arrangement has been selected for its ability to produce higher yields of fatty acids (additive effect) [6]. From the systematic inactivation of all ACP domains by mutagenesis it was clear that the presence of only one active ACP domain is sufficient to carry out PUFA biosynthesis, and that additional ACP domains increase the yield of PUFA with diminishing returns [6]. From that work it can be concluded that the primary advantage of the tandem ACP arrangements is an increase in the number of covalent tethers available for production.

Similar observations had been made previously in machineries for the biosynthesis of the polyketide antibiotic mupirocin. Some of the elongation modules in the mupirocin PKS contain multiple ACP domains and these have been shown to act either in parallel or in series [7]. However, no single ACP was identified as a bottleneck for production of mupirocin and neighboring ACP domains can compensate for deletion or inactivation of the tandem ACP domains, suggesting that ACP domains are functionally equivalent. More recently, the activity of multiple ACP domains in the biosynthesis of curacin in cyanobacteria was measured [8]. In that work it was shown that the contributions of individual ACP domains are additive and that a region C-terminal to the ACP domains was capable of enhancing ACP function in a biochemical assay. However, that work also showed that most individual ACP fragments had higher a molecular weight measured by gel filtration than the molecular weight indicated by sequence, raising the possibility that ACP domains may be forming higher order oligomers that stabilize the overall three-dimensional structure.

In this work we have investigated the structure of a tandem ACP domain fragment in solution. Our results show that the tandem ACP fragment is a monomer comprised of independent domains in an extended conformation.

## Results

### Determination of ACP Domain Boundaries Using the Udwary-Merski Algorithm

**Figure 3:**
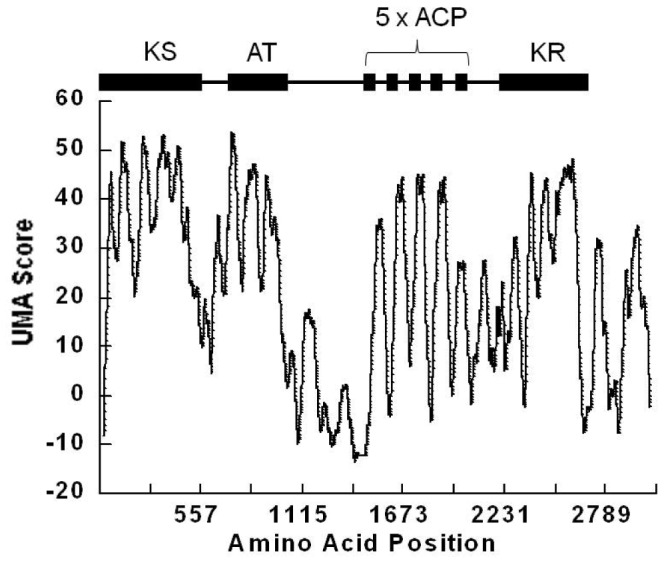
Domain structure prediction. The UMA method was employed to identify the ACP domains. The UMA score, a measurement of the likelihood that an amino acid is located within a domain (as opposed to within an unstructured linker) was plotted as a function of the position in the amino acid sequence. Five areas of high UMA score were identified as potential ACP fragments.

Initial attempts to identify the boundaries for ACP domains contained in the pfaA multienzyme of *Photobacterium profundum*, were carried out using the BLASTP tool (NCBI). The BLAST analysis yielded four segments of high similarity to ACP, corresponding to ACP 2,3,4 and 5. To further refine the determination of exact domain boundaries, we used the Udwary-Merski Algorithm (UMA) which assigns a score to each amino acid based on the probability that the amino acid is located within a domain or in an unstructured linker [9]. The UMA analysis of the pfaA sequence reveals areas of high score corresponding to one KS, one AT domain, five ACP domains and one KR domain (Figure 3). One of the five ACP domains detected by UMA, ACP1, had not been identified in the initial BLASTP search, thus highlighting the power of the UMA method in the identification of novel domains [10]. Another ACP domain, ACP5, was found to have a lower UMA score although it was identified in the BLASTP search, possibly suggesting that the fifth ACP domain may not be as conserved or may be more hydrophobic than the other domains. Interestingly, the UMA bioinformatic tool identified a region (residues H1771–R1791) with a high score but a low sequence similarity to ACP, potentially the C-terminal region which enhances biosynthetic activity as described in Gu *et al*., 2011 [8].

A fragment consisting of all five ACP domains (pfaA *Photobacterium profundum*: 1224–1735) was amplified by PCR and cloned into pET200Topo expression vector for production in *E. coli*. Additionally, the individual ACP domains that comprise the tandem ACP fragment were cloned and expressed as well. Typical yields for all ACP fragment in this study was 7 mg of pure protein per liter of liquid culture. All proteins were purified to homogeneity by a combination of nickel affinity chromatography and anion exchange chromatography.

**Figure 4:**
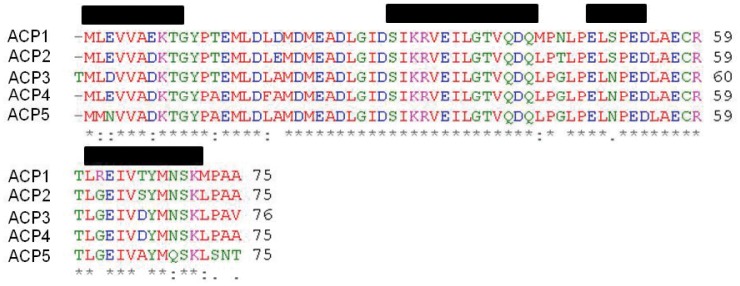
Multiple sequence alignment of the five ACP domains. The ACP domains from the *Photobacterium profundum* PUFA synthase were aligned using ClustalW. The black bars denote the stretches of protein sequence predicted to be α-helices.

A sequence alignment of the five ACP domains shows how nearly identical these ACP domains are (Figure 4). With only 13 amino acid positions which are not absolutely conserved for all five ACP domains, it is not entirely apparent why the first ACP domain would not have been recognized as such in a BLAST search, or why the fifth ACP domains would have a lower UMA score. The UMA algorithm consists of terms for secondary structure propensity, sequence conservation and hydrophobicity. Apparently, the sequence for the fifth ACP domain has a lower propensity for regular secondary structure than the other four domains.

### Verification of ACP Functional Competence by ESI-MS

**Figure 5:**
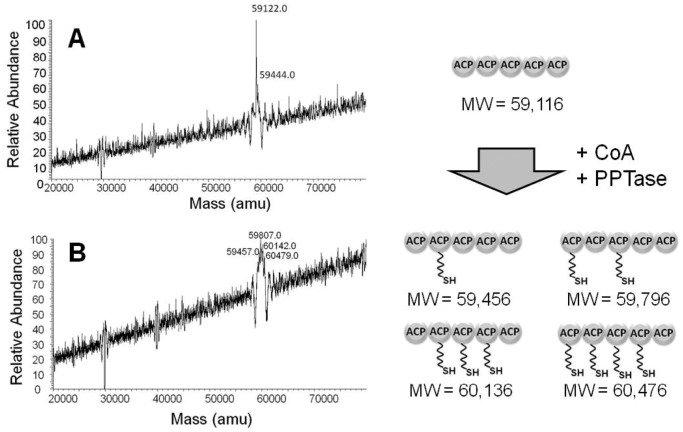
Characterization of ACP domains by mass spectrometry. (A) An ESI-MS spectrum was generated for the tandem ACP (MW  = 59,116) before modification by attachment of the phosphopantetheine moiety of CoA. (B) After incubation with PPTase and CoA the protein was modified in four attachment sites, as evidenced by the ESI-MS mass spectrum.

Functionally competent ACP domains are typically modified by the enzyme-catalyzed transfer of the 4′-phospho-pantetheinyl moiety from coenzyme A (CoA) [11]. In order to assess whether the recombinant ACP fragments are functionally competent, we incubated the purified ACP protein with the *sfp* phosphopantethinyl transferase (PPTase) from *Bacillus subtilis* and CoA, and the reaction products were analyzed by LC-MS to determine the molecular weight of the unmodified and modified ACP proteins. Most of the purified tandem-ACP is initially in an unmodified form as evidenced by the measured molecular weight of 59,122 Da, which is close to the molecular weight of 59,116 Da determined by the amino acid sequence (Figure 5A). However, there is a small portion of the protein which shows a change of +322 Da which suggests that some of the protein may have been modified by the endogenous PPTase from the *E. coli* expression host. The reaction products show a distribution of modified ACP domains in which either one, two, three or four ACP domains were modified as evidenced by their increased molecular weight (Figure 5B). No peak was detected corresponding to the tandem ACP modified on all five domains.

### Solution Structure of Tandem ACP by Small-angle X-ray Scattering (SAXS)

**Figure 6:**
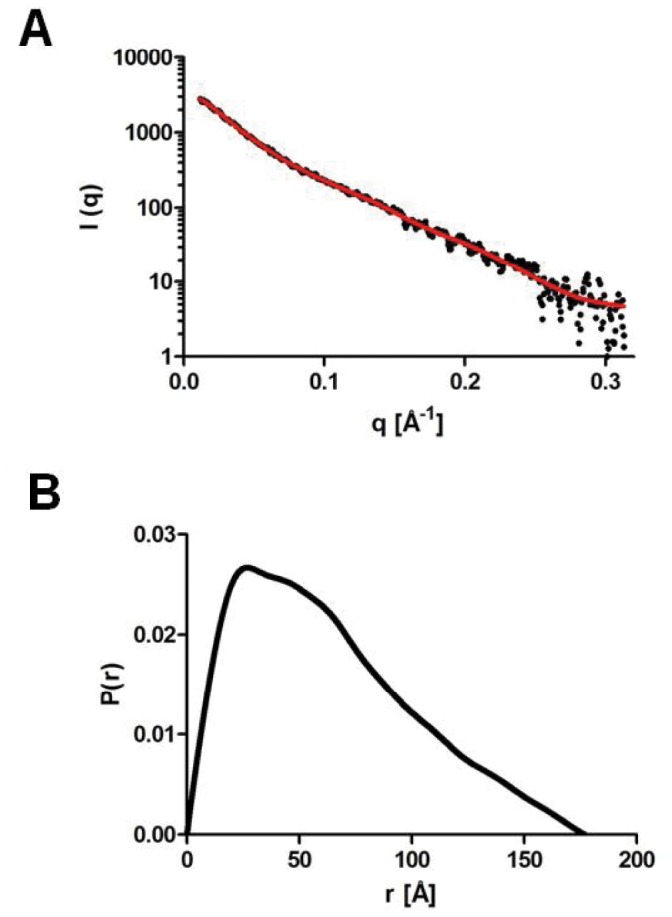
Small-angle X-ray Scattering Data. (A) Small X-ray Scattering (SAXS) Data for the tandem ACP fragment. (B) Calculation of the pair distribution function *P(r)* shows a shape compatible with an elongated structure, a radius of gyration (*Rg)* of 5.2±0.1 nm and a maximum diameter of 20±2 nm.

**Figure 7:**
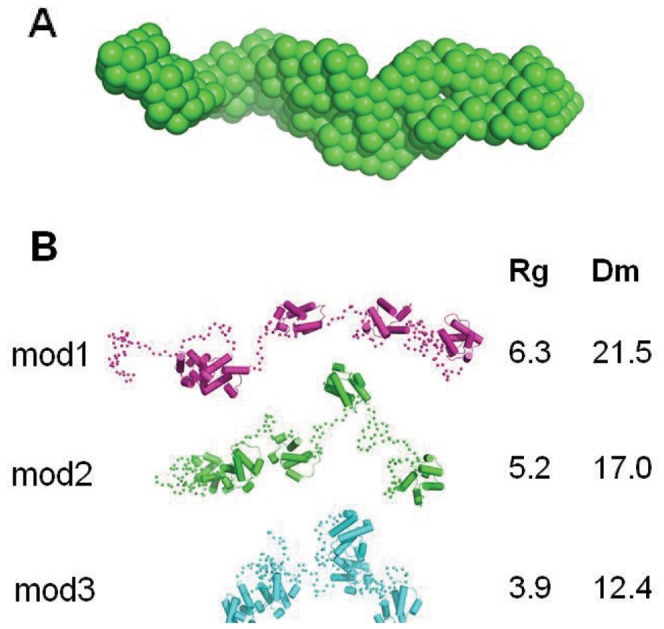
Solution structure of tandem ACP. (A) The three-dimensional bead model constructed by ‘dammif’ reveals a molecular volume of 96,600 Å^3^. (B) Simulation of the scattering data based on structural models reveals that an extended and flexible overall configuration can sufficiently account for the observed data. Figures A and B are on a different scale.

Small-angle X-ray scattering (SAXS) was used to generate a model for the solution structure of the tandem ACP. Analysis of the distance distribution (*P(r)*) of the signal resulted in an estimated radius of gyration of ∼5.2 nm and a maximum diameter (Dm) of ∼20 nm. The shape of *P(r)* is compatible with a rod-like elongated particle (Figure 6). The three-dimensional bead model constructed by ‘dammif’ reveals a molecular volume of 96,600 Å^3^, whereas the molecular (unhydrated) volume estimated from the three-dimensional models of the individual ACP domains is 90,400 Å^3^ (Figure 7A). This correspondence between the SAXS-derived volume and the volume calculated from the molecular models of the individual ACP domains strongly suggests that the tandem ACP is monomeric. In order to better understand the configurational variability or flexibility of the tandem ACP due to domain-domain interactions, we also used the ensemble optimization method (EOM) in which a mixture of different states was considered (Figure 7B). We obtained χ values of 1.144, 0.984, 0.971, 0.971, 0.968, 0.968 for ensembles constituted of 1-2-3-4-5 and 20 models respectively. The decrease of the χ value up to ensembles with 5 or more models is indicative of a flexible tandem arrangement, and speaks strongly against a compact quaternary arrangement of the ACP domains. The average Radius of gyration (*Rg*) of the selected 20-model ensemble (54 Å) was significantly larger than the average *Rg* of the 8000 generated models with random linker conformations (43 Å) indicating a tendency of the tandem construct to adopt an elongated arrangement. All of these data suggest that there are only a few interactions between the ACP domains consistent with a beads-on-a-string model in which the linkers are flexible and the domains are seemingly autonomous.

### Chromatographic Retention of Tandem and Individual ACP Domains

**Figure 8:**
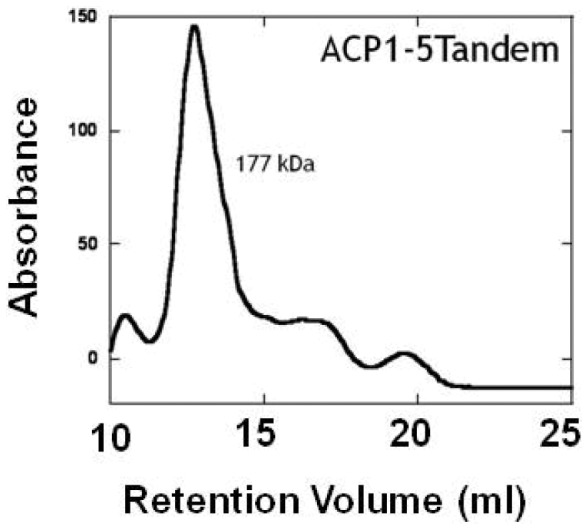
Size exclusion chromatography of tandem ACP. The tandem ACP analyzed on the same gel filtration column had the retention time of a protein three times its size (MW_est_  = 177,000).

When the tandem ACP (MW  = 59,116) was analyzed by gel filtration chromatography the estimated molecular weight (MW_est_ ≈ 177,000) based on its retention time, was more consistent with a trimeric domain than with a monomeric one (Figure 8). Similarly, when individual ACP domains were purified and analyzed by gel filtration, their estimated molecular weights were found to be more consistent with dimeric rather than monomeric domains (Table 1). However the Stokes Radius (*Rs*) of the tandem arrangement (48.6 Å), derived from analytical gel filtration, was very similar to the average *Rs* of the EOM ensemble determined by SAXS (47 Å). Thus the apparent trimeric state of tandem ACP can be explained by a shortened retention time of the monomer due to its extended conformation. Similarly, the apparent dimeric states of the individual ACPs are explained by their greater apparent size which is possibly due to the presence of the flexible linkers flanking each individual ACP.

**Table 1:** Expected and Measured Molecular Weights of ACP Constructs in Solution.

Construct	Expected MW (kDa)	Determined MW by gel filtration (kDa)
ACP_1_	16.134	34
ACP_2_	17.539	47
ACP_3_	17.523	39
ACP_5_	15.952	27
Tandem	59.116	176

### Thermal Denaturation of Tandem and Individual ACP Domains

**Figure 9:**
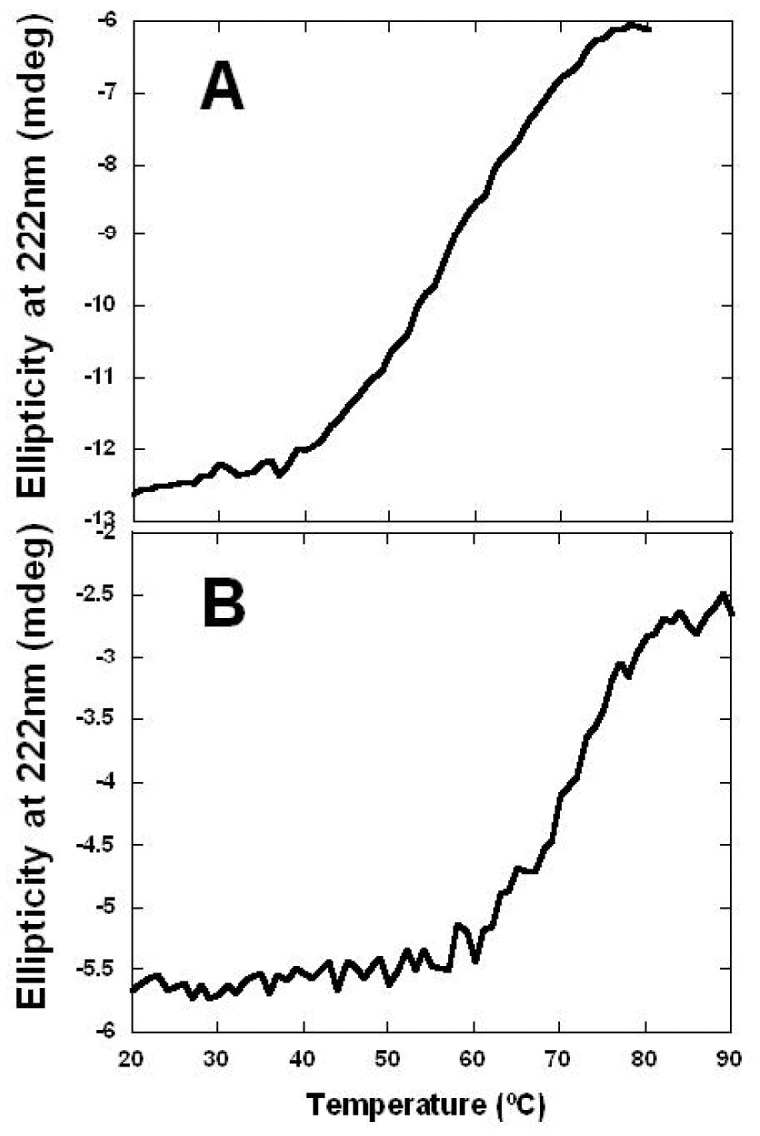
Thermal denaturation of individual and tandem ACP. Thermal denaturation of (A) the tandem ACP and (B) the individual ACP1 was followed by the loss of molar ellipiticity at 222 nm on a circular dichroism (CD) spectropolarimeter. The denaturation temperature (T_m_) for the tandem ACP was interpolated to be 59°C, whereas the individual ACP1 had a T_m_ of 71°C.

**Figure 10:**
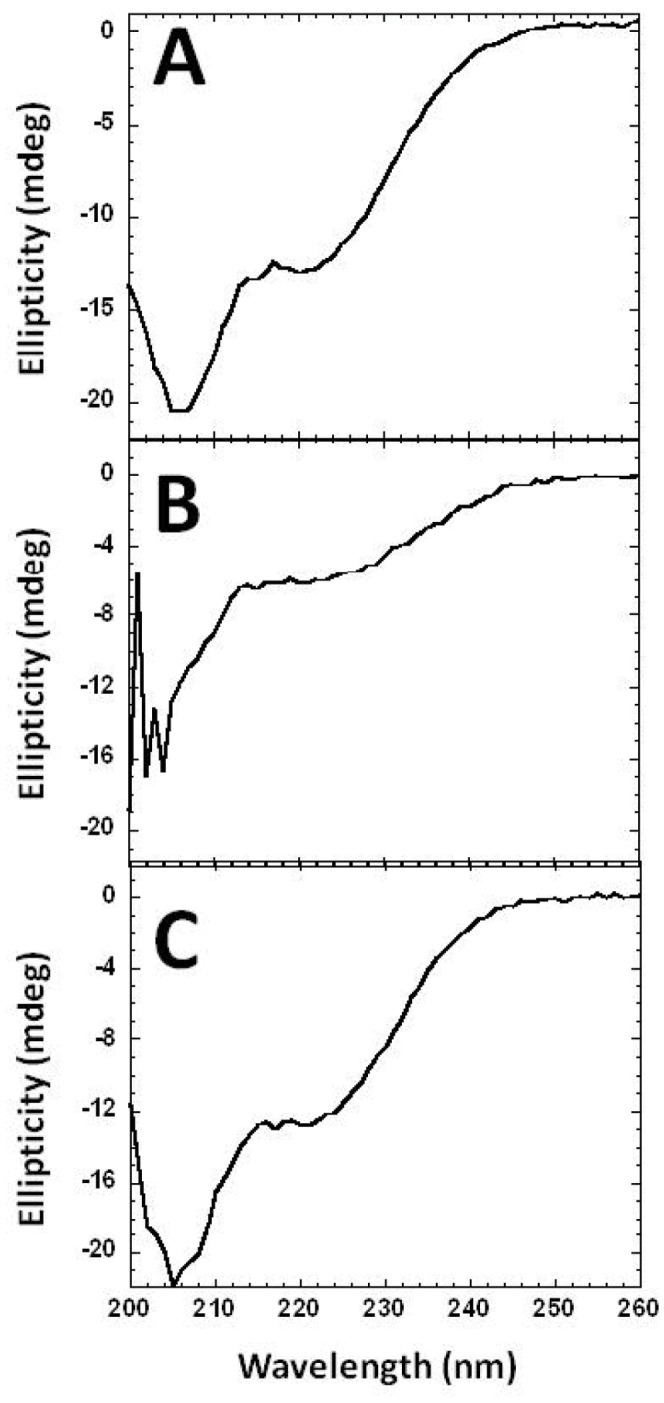
Full CD spectrum for the tandem ACP fragment obtained (A) before (B) during and (C) after thermal denaturation at 90°**C.**

In order to assess whether the tandem arrangement of ACP domains resulted in the overall stabilization of the protein compared to the individual domains, the thermal denaturation of both tandem and individual ACP domains was carried out by circular dichroism (CD) spectroscopy. The loss of molar ellipticity at 222 nm was measured as a function of temperature between 20–90°C (Figure 9). The denaturation temperature (T_m_) for the tandem ACP domains (59°C) was lower than for the individual ACP domain (71°C), indicating that the presence of multiple domains in tandem does not result in further stabilization of the protein (Figure 9). The unfolding of the tandem domains was not cooperative, speaking against stable domain-domain interactions. The thermal unfolding of tandem and individual ACP constructs was found to be non-cooperative and fully reversible as evidenced by the full recovery of the signal on successive temperature scans (Figure 10). Analysis of the CD data using the online program K2D2 for the determination of secondary structure composition, reveals a 38% helical content which is consistent with the 41% helical content encoded within the four-helix bundles of the ACP domains. Thus, the helical content of the tandem fragment can be fully accounted for by the ACP domains, with no contribution from the linker regions.

## Discussion

The biosynthesis of fatty acids and polyketides requires the presence of ACP domains either as independent proteins or embedded within elongation modules [12]. It has been reported that some ACPs in both land-based and marine organisms are arranged in tandem [13], [14]. Although no clear idea exists regarding the evolutionary advantages of a tandem ACP arrangement, much valuable research has aimed to characterize the functional consequences of such an arrangement (6–8). Although much is known about the structure of individual ACP domains by a variety of crystallographic and spectroscopic methods, the solution structure of a fragment consisting of multiple ACPs had not been explored [12], [15], [16]. In this work we have characterized for the first time, the solution structure of a protein fragment consisting of five tandem ACPs from a PUFA synthase from deep-sea bacteria. The resulting structural model based on small-angle X-ray scattering (SAXS) data, reveals a beads-on-a-string configuration with highly flexible and relatively independent domains.

Our initial gel filtration purification of the five-tandem-ACP fragment showed a shorter retention time than expected for a protein of its molecular weight, suggesting an oligomeric quaternary structure for the tandem fragment. Similarly, the individually expressed ACP domains also had chromatographic retention times that were short and consistent with dimeric ACPs. All of these results from gel filtration chromatography suggested the possibility of substantial association among the different ACP domains in the arrangement, thus providing additional intra-molecular stabilization for the overall structure of the PUFA synthase.

Similar observations of tandem ACP fragments with decreased chromatographic retention times have been reported recently [8]. In that work, fragments consisting of two or three tandem ACP domains from the curacin PKS were also found to have shorter retention times corresponding to ACP dimers, although the individual ACP domains had retention times closer to the expected value for the monomer.

Interestingly, the SAXS derived structural model in this report reveals a flexible elongated monomer, thus invalidating the possibility of intramolecular stabilization through the formation of a globular unit. The decreased chromatographic retention times are therefore the result of irregular hydrodynamics arising from its extended structure. Further support of the elongated beads-on-a-string tandem ACP model stems from the thermal denaturation experiments, which revealed that the tandem arrangement did not result in the stabilization of the overall protein structure and did not unfold cooperatively.

Others have suggested that the advantage of the tandem ACP arrangement in other multi-enzyme systems is that it builds redundancy by providing multiple functionally equivalent sites of synthesis [6], [7]. For examples, the PKS multi-enzyme responsible for the production of the antibiotic mupirocin in *Pseudomonas fluorescens* contains two sets of tandem ACPs which can function either in series or in parallel [7]. In that work it was reported that the deletion or inactivation of one of the tandem ACP domains from the mupirocin PKS caused a decrease, but not a total disruption, of mupirocin production. In Jiang *et al.*, the systematic inactivation of five (of the six) tandem ACPs from the PUFA synthase in *Shewanella japonica* resulted in a decrease, but not a total disruption, of PUFA production [6]. The inactivation of all six tandem ACPs from the PUFA synthase did abolish PUFA production. From these experiments it can be concluded that the consequence of ACP multiplication along the enzyme is that it provides additional sites of attachment for PUFA formation. Our SAXS-derived tandem ACP model is consistent with this proposed function of biosynthesis amplification since a beads-on-a-string ACP structure would allow for simultaneous access of the other enzyme domains which “service” them.

Modular or multidomain proteins are common in nature, but their structural characterization has posed a number of challenges [17]. These multi-domain proteins are often held together by unstructured linkers that render the protein too flexible for structural analysis by X-ray crystallography [18]. While nuclear magnetic resonance (NMR) provides a more appropriate method for structural analysis of flexible proteins, the large size of multi-domain assemblies is an impediment for NMR structure determination. On the other hand, small angle scattering (SAS) techniques, such as the one used in this study, are better suited to provide an overall topological model for the arrangement of domains in a flexible multi-domain arrangement in solution [19]. A number of multi-domain protein structures have been elucidated using SAS techniques thanks to the development of ensemble optimization methods that account for inherent structural flexibility [19]. In this work, we have used two different methods for the analysis SAXS data from a multi-domain ACP and both gave consistent results. The inherent flexibility of tandem ACP evidenced in the SAXS-derived structure indicates that crystallization of this construct is not possible. Additionally, the size of the tandem ACP (59,000 kDa) would have made the NMR analysis very difficult. Also, the high similarity between the individual ACP constituents of the tandem ACP (∼90% identity) would have made the resonance assignments nearly impossible. Thus, it is clear that the structural insight in this report could not have been obtained without the implementation of SAXS

The observation that linked ACP monomers can be used independently by biosynthesis machineries could have implications in biotechnology. It is conceivable that new strategies for maximizing the production of fatty acids and polyketide natural products will involve the construction of artificially tandem ACP domains to amplify production.

## Materials and Methods

### Materials and General Methods

All reagents such as kanamycin, chloramphenicol, ampicillin DNase, coenzyme A, MgCl2, IPTG (isopropyl-b-D-thiogalactopyranoside), dithiothreitol (DTT), glutathione, yeast extract, NaCl, tryptone, Nickel-NTA agarose and glutathione agarose were purchased from Sigma. All restriction endonucleases, polymerases and molecular biology reagents were purchased from New England Biolabs. Purification of DNA from agarose gels was performed using the QIAQuick Gel extraction kit (QIAgen).

### UMA Analysis of the pfaA Sequence

The UMA program was kindly provided by Craig Townsend (Johns Hopkins University) and Daniel Udwary (University of Rhode Island). UMA calculations were done as in Udwary et al., 2002 [9] using the sequence of pfaA from Photobacterium profundum (GenBank Accession no. AF409100.1). A multiple alignment of homologues of pfaA was performed in CLUSTALW in “.pir” format and a secondary structure prediction for pfaA was performed using the PSIPRED Server (University College London). The output for the secondary structure prediction was used to generate an “.ss” file. Finally, both the “.pir” alignment and the “.ss” secondary structure prediction were used as inputs for the uma19.pl application with input parameters which are summarized in Table 2. Results in the output file were visualized using Kaleidagraph for Windows.

**Table 2:** Input parameters for the UMA calculations.

Parameter	Value
Homology matrix	blosum 30
Gap to gap penalty	0
Gap to aa penalty	−4
Component averaging (k)	5
Final averaging (gamma)	20
Sim score weight	10
Struc score weight	1
Hydro score weight	5

### Cloning, Expression and Purification of ACP Domains

The DNA encoding the ACP fragments from pfaA was amplified from fosmid 8E1, which was originally described by Allen and Bartlett, 2002 and kindly provided by Dr. Eric Allen from the Scripps Institution of Oceanography [2]. The oligonucleotides used for the polymerase chain reaction (PCR) amplification of ACP fragments are summarized in table 3. The resulting amplicons were cloned into pET200Topo by following the TOPO Cloning Protocol (Invitrogen). Cloning was confirmed by DNA sequencing. E. coli BL21(DE3)-Codon Plus RIL cells was transformed with plasmids harboring the different ACP constructs and grown in liquid Luria-Bertani medium which contained kanamycin (100 mg/L) and chloramphenicol (25 mg/L) at 37°C, 325 rpm until A_600_ = 0.6 at which time protein expression was induced with 1 mM IPTG and the temperature was decreased to 22°C. After 16 h, the cells were harvested by centrifugation at 4°C and resuspended in in lysis buffer (50 mM Tris, pH 8.0, 150 mM NaCl, 1 mM DTT, 10% Glycerol) in the presence of lysozyme (1 mg/ml), DNase (4 µg/ml) and sonicated. The lysate was collected and poured through a column filled with Ni-NTA resin equilibrated in 50 mM Tris pH 8.0, 150 mM NaCl, 1 mM DTT and 10% glycerol. The ACP fragments were eluted with the same equilibration buffer but containing 300 mM imidazole. The protein eluate was concentrated and injected onto a Hi Load 16/10 Q Sepharose column (GE Healthcare) equilibrated in buffer A (50 mM Tris pH 8.0, 150 mM NaCl, 1 mM DTT and 10% glycerol). The protein was eluted using a linear NaCl gradient for 0% to 100% buffer B (50 mM Tris pH 8.0, 2 M NaCl, 1 mM DTT and 10% glycerol). The purity was ascertained by SDS-PAGE electrophoresis. Typical protein yields were 7 mg of pure protein per liter of culture.

**Table 3:** Oligonucleotides used for the amplification of the different proteins in this study.

Constructs	Oligonucleotide sequences
ACP1-Fwd	5′- CACCGCACCACAAGCAGCAGTTCAAACT-3′
ACP1-Rev	5′-TGCTGCGTCTAAGCCGTTTGATGC-3′
ACP2-Fwd	5′-CACCTCAGCATCAAACGGCTTAGA-3′
ACP2-Rev	5′-TGCTGACGCGACTGGAGCAGAA-3′
ACP3-Fwd	5′-CACCGCGTCAGCATCAAACGGTTT-3′
ACP3-Rev	5′-TGGAGCAGCTGTTTGAATCGCTAC-3′
ACP4-Fwd	5′-CACCGCATCAAACGGTTTAGATGC-3′
ACP4-Rev	5′-GTTGCTACTGGTGCTGCTACTGT-3′
ACP5-Fwd	5′-CACCTCCTGCAACAAACGGCTTAG-3′
ACP5-Rev	5′-CAAGGTTTTCAACAGCAGGTGCAG-3′
ACP1-5Tandem-Fwd	5′-CACCGCACCACAAGCAGCAGTTCAAACT-3′
ACP1-5Tandem-Rev	5′-CTACAAGGTTTTCAACAGCAGGT-3′
Sfp-Fwd	5′-CATGCATGGGATCCATGAAGATTTACGGAATTTATATGGA-3′
Sfp-Rev	5′-CATGCATGCCCGGGCTATATAAAAGCTCTTCGTACGAGACC-3′

### Cloning, Expression and Purification of *sfp* PPTase

The cloned gene for Sfp from Bacillus Subtilis (Sfp:pET28b) was a generous gift from Dr. Kira Weissman (University of Saarland, Germany). The gene for Sfp was amplified as described for the ACP fragments. The amplicon was ligated via blunt-end cloning into pUC19. The resulting plasmid was digested with SmaI and BamHI restriction endonucleases and the sfp fragment was cloned into the pGEX4T-3 plasmid (GE Healthcare). E. coli BL21(DE3)-Codon Plus RIL cells was transformed with the sfp:pGEX4T-3 plasmid and grown in liquid Luria-Bertani medium which contained ampicillin (100 mg/L) and chloramphenicol (25 mg/L) at 37°C, 325 rpm until A_600_ = 0.6 at which time protein expression was induced with 1 mM IPTG. After 16 h, the cells were harvested by centrifugation at 4°C and resuspended in in lysis buffer (50 mM Tris, pH 8.0, 150 mM NaCl, 1 mM DTT, 10% Glycerol) in the presence of lysozyme (1 mg/ml), DNase (4 µg/ml) and sonicated. The lysate was collected and poured through a column filled with glutathione agarose equilibrated 50 mM Tris pH 8.0, 150 mM NaCl, 1 mM DTT and 10% glycerol. GST-fused sfp was eluted in the same buffer containing 10 mM reduced glutathione (Sigma) and dialyzed for 16 hours at 4 oC into a buffer containing 11.8 mM Tris, pH 8.0, 143 mM NaCl and stored in 20% glycerol.

### ESI-MS of Recombinant ACP Domains

Purified ACP1 was exchanged into 10% Acetonitrile in water using a Vivaspin concentrator (MWCO 3000) and diluted in a solution containing acetronitrile:water (50∶50 vol/vol) and 0.2% formic acid. The diluted protein was directly infused in the nano-electrospray source at 2 µM. The ionization source was set at a spray voltage of 1.5kV and capillary temperature 250°C. The ionized molecules were analyzed in a LTQ mass spectrometer (Thermo Fisher Scientific). The mass spectra were recorded for 1 minute. The data acquired were then analyzed using BioMass calculation and deconvolution tool of the BioWorks 3.1 software. The parameters for BioMass calculation and deconvolution were set as follows: enable averaging, enable smoothing (Gaussian; 3), deconvoluted spectrum (10,000–20,000), adduct ion mass [+ proton (+1.008)], and mass step size 0.25 units.

### Gel Filtration Chromatography

The purified proteins were loaded onto a HiPrep Superdex 200 10/300 GL column (GE Healthcare) equilibrated in 50 mM sodium phosphate pH 7.2, 150 mM NaCl, 1 mM DTT. Each run with the ACP proteins was preceded by a run with a mixture of standard proteins [ferritin (440,000 Da), aldolase (158,000 Da), conalbumin (75,000 Da), carbonic anhydrase, (29,000 Da), ribonuclease A (13,700 Da), and apropotin (6,500 Da)]. The Stokes radii of these proteins standards were ferritin (Rs  = 61 Å), aldolase (Rs  = 48.1 Å), conalbumin (Rs  = 36.4 Å), carbonic anhydrase (Rs  = 23 Å), ribonuclease (Rs  = 16.4 Å).

### Circular Dichroism Spectroscopy (CD)

A JASCO J-810 spectropolarimeter was utilized to collect the CD spectra of 5 µM Tandem and individual ACP5 at 25°C. The optical chamber (1 mm optical path length) was deoxygenated with dry purified nitrogen before use and kept in the nitrogen atmosphere during experiments. Multiple scans from 200 to 260 nm were accumulated and averaged. In each case, the background of the buffer solution was subtracted from the CD data. Thermal unfolding experiments were carried out by following CD at 222 nm as a function of a temperature range from 20–95°C. The melting curves were recorded by a UV spectrometer equipped with a temperature-controlled water bath, with a rate of 1°C/min. Both unfolding (from 20°C to 95°C) and refolding (from 95°C to 20°C) data points were collected and plotted using normalized absorbance versus temperature. The unfolding transitions were approximately 100% reversible; aggregation was never observed. All experiments were performed in a buffer containing 10 mM HEPES, 150 mM NaCl, 1 mM TCEP-HCl, pH 7.5. Care was taken to let all buffer/protein mixtures equilibrate before measurements. The CD data was analyzed to determine secondary structure composition using the K2D2 online program [20].

### Small Angle X-ray Scattering

Tandem ACP was buffer exchanged into 10 mM Hepes pH 7.5, 150 mM NaCl, 1 mM EDTA, 1 mM TCEP, 5 mM β-mercaptoethanol and concentrated to 1.5 mg/ml. Data were recorded at the SIBYLS beamline 12.3.1. at the Advanced Light Source at the Lawrence Berkeley National Laboratory, Berkeley, CA, at 10 °C using a wavelength of 1 Å. Data were recorded for values of the momentum transfer vector *q* = (4πsinθ)/λ between 0.01 and 0.32 Å^−1^. The same sample was sequentially exposed to x-rays for 0.5s, 1s and 5s. Samples containing buffer only were measured before and after protein samples. The buffer contributions were subtracted from protein scattering data using the ogreNew program available at SIBYLS. Data were checked for radiation damage as indicated for a higher apparent *Rg* of the Guinier region for subsequent sample exposures. A combined SAXS pattern was obtained through scaling and merging selected regions of buffer-subtracted scattering pattern for the 0.5s, 1s and 5s exposures (using both buffer-subtracted data sets of repeat experiments). The resulting combined and merged scattering pattern was analyzed using PRIMUS, SASREF,DAMMIF and EOM of the ATSAS software package [21] using a resolution range of *q* = {0.01–0.32 Å^−1^}. The *Rg* value derived from the Guinier analysis (5.0±0.1 nm) corresponded well to the *Rg* obtained through the indirect transform algorithm in GNOM (5.2±0.1 nm). For EOM analysis, homology models of the individual ACP domains were constructed using the Swiss-Model web server [22] based on the ACP sequences identified in this work. ACP 1 - 5 were modeled based on PDB entry 2EHT (ACP from Aquifex aeolicus).
